# *Bacillus subtilis* Nucleoid-Associated Protein YlxR Is Involved in Bimodal Expression of the Fructoselysine Utilization Operon (*frlBONMD-yurJ*) Promoter

**DOI:** 10.3389/fmicb.2020.02024

**Published:** 2020-08-21

**Authors:** Mitsuo Ogura, Kazutoshi Shindo, Yu Kanesaki

**Affiliations:** ^1^Institute of Oceanic Research and Development, Tokai University, Shizuoka, Japan; ^2^Department of Food and Nutrition, Japan Women’s University, Tokyo, Japan; ^3^Research Institute of Green Science and Technology, Shizuoka University, Shizuoka, Japan

**Keywords:** amino sugar utilization, bimodal expression, autoregulation of *ylxR*, *Bacillus subtilis*, bet-hedging strategy

## Abstract

Bacteria must survive harsh environmental fluctuations at times and have evolved several strategies. “Collective” behaviors have been identified due to recent progress in single-cell analysis. Since most bacteria exist as single cells, bacterial populations are often considered clonal. However, accumulated evidence suggests this is not the case. Gene expression and protein expression are often not homogeneous, resulting in phenotypic heterogeneity. In extreme cases, this leads to bistability, the existence of two stable states. In many cases, expression of key master regulators is bimodal via positive feedback loops causing bimodal expression of the target genes. We observed bimodal expression of metabolic genes for alternative carbon sources. Expression profiles of the *frlBONMD-yurJ* operon driven by the *frlB* promoter (P*frlB*), which encodes degradation enzymes and a transporter for amino sugars including fructoselysine, were investigated using transcriptional *lacZ* and *gfp*, and translational fluorescence reporter *mCherry* fusions. Disruption effects of genes encoding CodY, FrlR, RNaseY, and nucleoid-associated protein YlxR, four known regulatory factors for P*frlB*, were examined for expression of each fusion construct. Expression of P*frlB-gfp* and P*frlB*-*mCherry*, which were located at *amyE* and its original locus, respectively, was bimodal; and disruption of *ylxR* resulted in the disappearance of the clear bimodal expression pattern in flow cytometric analyses. This suggested a role for YlxR on the bimodal expression of P*frlB*. The data indicated that YlxR acted on the bimodal expression of P*frlB* through both transcription and translation. YlxR regulates many genes, including those related to translation, supporting the above notion. Depletion of RNaseY abolished heterogenous expression of transcriptional P*frlB-gfp* but not bimodal expression of translational P*frlB*-*mCherry*, suggesting the role of RNaseY in regulation of the operon through mRNA stability control and regulatory mechanism for P*frlB*-*mCherry* at the translational level. Based on these results, we discuss the meaning and possible cause of bimodal P*frlB* expression.

## Introduction

Bacterial “collective” behaviors of single cells have evolved to adapt to their harsh environments and have been identified as a result of recent progress in single-cell analysis (Veening et al., 2008; Bury-Moné and Sclavi, 2017). A growing number of examples show these behaviors (Kröger et al., 2011; Afroz et al., 2014; Kotte et al., 2014; Solopova et al., 2014; Norman et al., 2015; Leh et al., 2017; Kampf et al., 2018; Weiss et al., 2019). Heterogeneous expression of genes and proteins often leads to phenotypic heterogeneity. In extreme cases, this results in bistability, the existence of two stable states in a single population. For example, in *Bacillus subtilis* development of genetic competence for uptake of extracellular DNA, bimodal expression of the key master regulator ComK is observed, which leads to the differentiation of a fraction of cells among the cell population into the competent state (Maamar and Dubnau, 2005; Dubnau and Losick, 2006). When it comes to heterogeneous expression systems, bacteria sometime adopt a “bet-hedging strategy” where they differentiate into subpopulations in the same culture in order to facilitate adaptation to rapid environmental fluctuations (Veening et al., 2008; Norman et al., 2015). In this strategy, the cells to be adapted for the fluctuation with different phenotype have differentiated from sibling cells even before the environmental change. For example, *Bacillus* sporulation can be regarded as “bet-hedging” since the sporulating subpopulation prepares for more nutritionally harsh environments while the non-sporulating subpopulation retains the chance to re-initiate cell growth if more nutrients become available. In this case, highly heterogeneous phosphorylation of the master sporulation regulator Spo0A triggers the initiation of sporulation (Chastanet et al., 2010).

**FIGURE 1:**
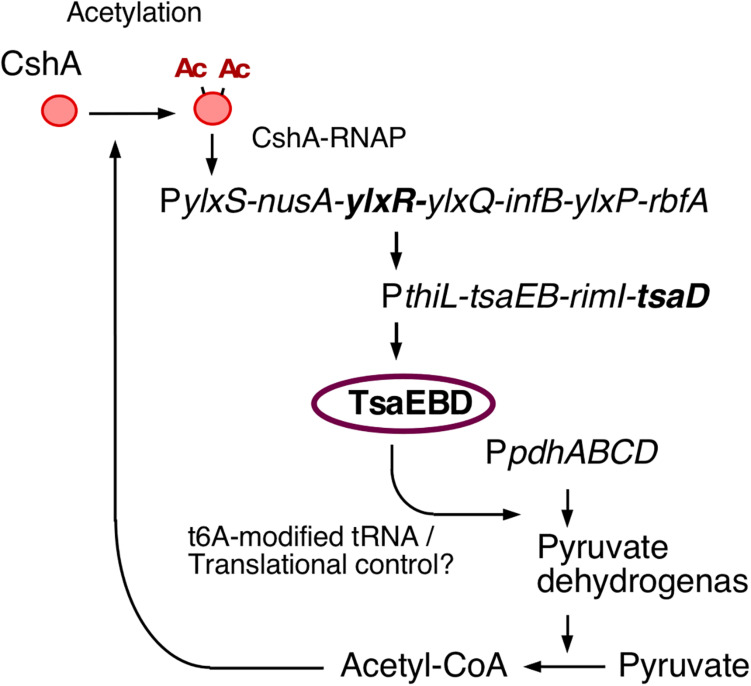
The indirect positive feedback loop of P*ylxS* expression. The indicated pathways were previously identified: *in vivo* association of CshA with RNAP (Delumeau et al., 2011), CshA acetylation (Kosono et al., 2015; Ogura and Asai, 2016), and CshA-dependent P*ylxS* regulation driving YlxR expression, which regulates transcriptional regulation of *tsaEBD* through the functional YlxR-binding to the promoter of *tsaEBD*, whose products are assembled and regulate pyruvate dehydrogenase translation (Ogura et al., 2019). Pyruvate dehydrogenase provides acetyl-CoA, which would be the acetyl moiety source for CshA acetylation. Arrows indicate transcription, translation, acetylation, enzymatic reaction, transcriptional activation, or metabolic reaction. Ac, acetyl moiety; RNAP, RNA polymerase.

YlxR has characteristics specific to nucleoid-associated proteins (NAPs) and exhibits non-homogeneous expression (Browning et al., 2010; Ogura and Kanesaki, 2018). The heterogeneous expression of YlxR was revealed through microscopic observation of green fluorescent protein (GFP) expression by an YlxR–GFP fusion, although the biological consequence remains unclear (Ogura and Kanesaki, 2018). YlxR regulates transcription of more than 400 genes, including many metabolic genes (Ogura and Kanesaki, 2018). For example, in a *B. subtilis ylxR*-deletion mutant, expression of the *frlBONMD-yurJ* operon for amino sugar utilization and two arginine biosynthetic operons were enhanced (Deppe et al., 2011a, b). Furthermore, YlxR positively regulated the *tsaEBD*-containing operon through direct binding of YlxR to the operon promoter (Ogura et al., 2019; Figure 1). TsaEBD is an enzyme required for the synthesis of threonylcarbamoyl adenosine (t^6^A)-modified tRNA (Thiaville et al., 2015; Zhang et al., 2015). The t^6^A-modified tRNA is conserved in all domains of life, and its deficiency sometimes causes severe dysfunctions (Thiaville et al., 2016; Ogura et al., 2019). Expression of the *ylxR*-containing operon driven by the *ylxS* promoter (P*ylxS*) itself requires *cshA* encoding a DEAD-box RNA helicase (Lehnik-Habrink et al., 2013; Ogura and Kanesaki, 2018). Proteomic analysis of *B. subtilis* revealed that CshA is lysine-acetylated (Kosono et al., 2015; Ogura and Asai, 2016). It has been reported that CshA associates with RNA polymerase (RNAP) and CshA-associated RNAP alters some of its own properties, such as its affinity to several sigma factors (Delumeau et al., 2011; Ogura and Asai, 2016). CshA acetylation is susceptible to pyruvate dehydrogenase (PDH) mutations in *pdhABCD* (Gao et al., 2002; Ogura and Asai, 2016). Disruption of the *pdh* genes reduces the intracellular acetyl-CoA pool and flux as a result of the loss of PDH activity, that is, the conversion of pyruvate to acetyl-CoA (Ogura et al., 2019). In *B. subtilis*, several lines of evidence suggest a relationship between low t^6^A and protein quality control, including PDH (Thiaville et al., 2015; Ogura et al., 2019). Therefore, t^6^A is required for a stable acetyl-CoA supply through control of PDH activity. Consequently, YlxR and CshA are concomitantly involved in the complex regulatory loop.

The *frlBONMD-yurJ* operon is driven by P*frlB*, which encodes metabolic enzymes fructoselysine-6-P-glycosidase from *frlB* and fructosamine kinase from *frlD*, and the FrlMNO-YurJ transporter for amino sugars, including fructoselysine. In the current study, expression profiles of the *frlBONMD-yurJ* operon were investigated using four fusion constructs. Two were transcriptional *lacZ* and *gfp* fusions at the ectopic locus *amyE*. The third was a transcriptional *gfp* fusion located at its original chromosomal location. The fourth fusion construct was a translational *mCherry* fusion at its original locus. Disruption effects of “genes encoding” CodY, FrlR, and YlxR, three known transcription factors for P*frlB*, were examined for expression of each fluorescent reporter fusion in flow cytometric analyses (Molle et al., 2003; Belitsky and Sonenshein, 2008; Deppe et al., 2011b). The two *gfp* fusions showed heterogeneous expression profiles. The expression of P*frlB-mCherry* was bimodal, but disruption of *ylxR* resulted in the disappearance of its clear bimodal expression. RNaseY is known to likely degrade mRNA of the *frlBONMD-yurJ* operon (Lehnik-Habrink et al., 2011), and depletion of the RNaseY-encoding gene resulted in abolishment of the heterogeneous expression of P*frlB-gfp*, but not bimodal one of P*frlB-mCherry.* These findings suggested a translational level of P*frlB* regulation and a role for YlxR on the bimodal expression of P*frlB*. Finally, we discuss the implications and possible causes of the bimodal expression of P*frlB*.

## Materials and Methods

### Strains, Media, and PCR

All *Bacillus subtilis* strains used in this study are shown in Table 1. One-step competence medium (MC) (Kunst et al., 1994), Schaeffer’s sporulation medium (SM) (Schaeffer et al., 1965), Luria–Bertani (LB Lennox) medium (Difco, MI, United States), and Antibiotic medium 3 (Difco) were used. Antibiotic concentrations were described previously (Ogura and Tanaka, 1996; Ogura et al., 1997). Synthetic oligonucleotides were commercially prepared by Tsukuba Oligo Service (Ibaraki, Japan) and are listed in Supplementary Table S1. For PCR-mediated construction of strains and plasmids, PrimeSTAR MAX DNA polymerase (Takara Co., Shiga, Japan) was used. For screening of recombinant DNA during plasmid construction, LA PCR DNA polymerase (Takara Co.) was used.

**TABLE 1:** Strains and plasmids used in this study.

**Strain**	**Genotype**	**References**
168	*trpC2*	Laboratory stock
OAM913	*trpC2 frlR*::Km^r^	This study
OAM816	*trpC2 ylxR*::Tn (Km^r^)	Ogura and Kanesaki, 2018
KK21	*trpC2 codY*(Cm^r^)	Hayashi et al., 2006
GP193	*trpC2 rny* (P*xylA*-*rny* Cm^r^)	Lehnik-Habrink et al., 2011
OAM-N32	*sinI*::psinI-SD-*gfp* (Cm^r^)	Ogura, 2016
OAM914	*trpC2 amyE*::P*frlB-lacZ* (Cm^r^)	This study
OAM915	*trpC2 amyE*::P*frlB-lacZ* (Cm^r^) *ylxR*(Km^r^)	This study
OAM916	*trpC2 amyE*::P*frlB-lacZ* (Cm^r^) *frlR*(Km^r^)	This study
OAM917	*trpC2 amyE*::P*frlB-lacZ* (Cm^r^)	This study
	*codY*(Cm^r^::Tc^r^)	
OAM933	*trpC2 amyE*::P*acsA-lacZ* (Cm^r^)	This study
OAM934	*trpC2 amyE*::P*codV-lacZ* (Cm^r^)	This study
FU676	*trpC2 amyE*::P*ilvB-lacZ* (Cm^r^)	Tojo et al., 2004
OAM818	*trpC2 amyE*::P*ylxS-gfp* (Cm^r^)	Ogura and Kanesaki, 2018
OAM938	*trpC2 amyE*::P*ylxS-gfp* (Cm^r^) *cshA* (Km^r^)	This study
OAM939	*trpC2 amyE*::P*ylxS-gfp* (Cm^r^) *ylxR*(Km^r^)	This study
OAM918	*trpC2 amyE*::P*frlB-gfp* (Cm^r^)	This study
OAM919	*trpC2 amyE*::P*frlB-gfp*(Cm^r^) *frlR*(Km^r^)	This study
OAM920	*trpC2 amyE*::P*frlB-gfp* (Cm^r^) *codY*(Cm^r^::Tc^r^)	This study
OAM921	*trpC2 amyE*::P*frlB-gfp* (Cm^r^) *ylxR*(Km^r^)	This study
OAM940	*trpC2 amyE*::P*frlB-gfp* (Cm^r^::Tc^r^) *rny* (Cm^r^)	This study
OAM935	*trpC2 amyE*::P*acsA-gfp* (Cm^r^)	This study
OAM936	*trpC2 amyE*::P*codV-gfp* (Cm^r^)	This study
OAM937	*trpC2 amyE*::P*ilvB-gfp* (Cm^r^)	This study
OAM922	*trpC2* P*frlB-gfp*(Cm^r^)	This study
OAM923	*trpC2* P*frlB-gfp*(Cm^r^) *frlR*(Km^r^)	This study
OAM924	*trpC2* P*frlB-gfp* (Cm^r^) *codY*(Cm^r^::Tc^r^)	This study
OAM925	*trpC2* P*frlB-gfp* (Cm^r^) *ylxR*(Km^r^)	This study
OAM926	*trpC2* P*frlB-gfp* (Cm^r^) *codY*(Cm^r^::Tc^r^) *ylxR* (Km^r^)	This study
OAM941	*trpC2* P*frlB-gfp* (Cm^r^::Tc^r^) *rny* (Cm^r^)	This study
OAM927	*trpC2* P*frlB-mCherry* (Cm^r^)	This study
OAM928	*trpC2* P*frlB-mCherry* (Cm^r^) *frlR*(Km^r^)	This study
OAM929	*trpC2* P*frlB-mCherry* (Cm^r^) *codY*(Cm^r^::Tc^r^)	This study
OAM930	*trpC2* P*frlB-mCherry* (Cm^r^) *ylxR*(Km^r^)	This study
OAM841	*trpC2 proB-lacZ* (Tc^r^) *ylxR*(Km^r^) *amyE*::P*xyl*-*ylxR* (Cm^r^)	Ogura and Kanesaki, 2018
OAM944	*trpC2* P*frlB-mCherry* (Cm^r^) *ylxR*(Km^r^) *amyE*::P*xyl*-*ylxR* (Cm^r^::Tc^r^)	This study
OAM931	*trpC2* P*frlB-mCherry* (Cm^r^) *codY*(Cm^r^::Tc^r^) *ylxR* (Km^r^)	This study
OAM942	*trpC2* P*frlB-mCherry* (Cm^r^::Sp^r^) *rny* (Cm^r^)	This study
OAM943	*trpC2* P*frlB-mCherry* (Cm^r^::Sp^r^) *rny* (Cm^r^) *ylxR* (Km^r^)	This study
OAM817	*trpC2 amyE*::*PylxS-gfp* (Cm^r^)	Ogura and Kanesaki, 2018
OAM932	*trpC2* P*frlB-mCherry* (Cm^r^) *amyE*::P*ylxS-gfp* (Cm^r^::Tc^r^)	This study
**Plasmid**	**Description**	
pIS284	Ampicillin resistance, *amyE*::*lacZ*(Cm^r^)	Tsukahara and Ogura, 2008
pIS284-frlB	Ampicillin resistance, *amyE*::P*frlB*-*lacZ*(Cm^r^)	This study
pIS284-acsA	Ampicillin resistance, *amyE*::P*acsA*-*lacZ*(Cm^r^)	This study
pIS284-codV	Ampicillin resistance, *amyE*::P*codV*-*lacZ*(Cm^r^)	This study
ECE75	Ampicillin resistance, Cm^r^::Tc^r^	BGSC
ECE73	Ampicillin resistance, Cm^r^::Sp^r^	BGSC
pUKM504	pUC19 carrying Amp^r^::Km^r^	Ogura and Tanaka, 1996
pUKM504-frlR	pUC19 carrying a part of *frlR* and Amp^r^::Km^r^	This study
pSG1194	Ampicillin resistance, *dsRed* (Cm^r^)	Feucht and Lewis, 2001
pfrlB-SD-gfp	pSG1194 carrying P*frlB-*SD*-gfp* instead of *dsRed*	This study
pNG621	Ampicillin resistance, *mCherry* (Cm^r^)	Doherty et al., 2010
pfrlB-mCherry	pNG621 carrying P*frlB*-*mCherry*	This study

### Plasmid Construction

The plasmids used in this study are listed in Table 1. For PCR, *B. subtilis* chromosomal DNA was used as template. To construct pIS284-frlB, pIS284-acsA, and pIS284-codV, PCR products were amplified using the oligonucleotides pairs pIS-frlB-F-E/pIS-frlB-R-B, pIS-acsA-F-E/pIS-acs-R-B, and pIS-codV-Eco/pIS-codV-Bam; digested with *Eco*RI/*Bam*HI; and cloned into pIS248 treated with the same enzymes (Tsukahara and Ogura, 2008). To construct pUKM504-frlR, PCR products were amplified using the oligonucleotides pair pUKM-frlR-B/pUKM-frlR-H, digested with *Bam*HI/*Hin*dIII, and cloned into a pUKM504 plasmid treated with the same enzymes (Ogura and Tanaka, 1996). To construct pfrlB-SD-gfp, oligonucleotide pairs pIS-frlB-F-E/PfrlB-(SD)-gfp-R and gfp(SD)-F/gfp-Xba-R were used for amplification of the genomic region and *gfp* from genomic DNA of strains 168 and OAM-N32, respectively (Ogura, 2016). Here, SD means Shine–Dalgarno sequence. After the combination of the both PCR products in the PCR using oligonucleotide pair pIS-frlB-F-E/gfp-Xba-R, the fragment digested with *Eco*RI/*Xba*I was cloned into pSG1194 without *dsRed* generated by digestion with the same restriction enzyme pair (Feucht and Lewis, 2001). To construct pfrlB-mCherry, PCR products were amplified using the oligonucleotide pair mChe-frlB-F-H/mChe-frlB-R-E, digested with *Hin*dIII and *Eco*RI, and cloned into pNG621 treated with the same enzymes (Doherty et al., 2010).

### Strain Construction

To construct OAM914, OAM933, and OAM934, the plasmids pIS284-frlB, pIS284-acsA, and pIS284-codV were transformed to the wild-type (WT) strain 168; and among the resultant chloramphenicol resistant colonies, those with amylase non-producing phenotype were selected on the LB agar plate containing 1% starch. To construct OAM913, OAM922, and OAM927, the plasmids pUKM504-frlR, pfrlB-SD-gfp, and pfrlB-mCherry were transformed to 168. To construct strains carrying the *amyE*:P*frlB*-*gfp*, *amyE*:P*acsA*-*gfp*, *amyE*:P*codV*-*gfp*, and *amyE*:P*ilvB*-*gfp* fusions, first, PCR products were amplified from strain OAM914 carrying *amyE*:P*frlB*-*lacZ*, OAM933 carrying *amyE*:P*acsA*-*lacZ*, OAM934 carrying *amyE*:P*codV*-*lacZ*, and FU676 carrying *amyE*:P*ilvB*-*lacZ*, using the oligonucleotide pairs amyE-RR/PfrlB-(SD)-gfp-R, amyE-RR/PacsA-(SD)-gfp-R, amyE-RR/PcodV-(SD)-gfp-R, and amyE-RR/PilvB-(SD)-gfp-R, respectively. Second, PCR products were amplified from the strain OAM-N32 using the oligonucleotide pair gfp(SD)-F/amyE-FF (Ogura, 2016). Each fragment for the promoters and the amplified *gfp-amyE* cassette was combined in a final PCR using the oligonucleotide pair amyE-FF/amyE-RR. The final PCR products were transformed into *B. subtilis* 168, and the chromosomal structure of the transformant was verified by PCR analysis using appropriate primers.

### β-Galactosidase Analysis

Growth conditions and β-galactosidase analysis procedures were previously described (Ogura and Asai, 2016; Ogura and Kanesaki, 2018).

### Microscopic Observations

Cells were picked up from flesh colony on LB agar plate and inoculated to 1 ml of LB medium in L-tube. The tube was shaken for 14 h at 37°C. One hundred microliters of the culture was centrifuged, and 80 μl of the supernatant was removed. The cells were then resuspended in the remaining 20 μl. Portions (2 μl) of each sample were mounted on glass slides treated with 0.1% (wt/vol) poly-L-lysine (Sigma-Aldrich, MO, United States). Microscopy was performed with an Olympus BX51 phase contrast and fluorescence microscope with a 100 × PLAN-N objective (Olympus, Tokyo, Japan). Images were captured using a CoolSNAP HQ charge-coupled device camera (Nippon Roper, Tokyo, Japan) and Metavue 4.6r8 software (Universal Imaging, PA, United States).

### Flow Cytometry Analysis

All the strains were streaked on LB agar plates supplemented with specific antibiotics and incubated overnight. The resulting single colony was picked up and grown overnight in 1 ml of LB medium in L-tube at 37°C. Cells were washed and resuspended in phosphate-buffered saline (PBS) and directly measured on BD LSRFortessa (Becton–Dickinson, CA, United States) with an argon laser (488 nm) and yellow green laser (561 nm). For each sample, the green fluorescent signal or mCherry signal of 30,000 cells was collected by a bandpass (BP) filter (530/30 nm, 610/20 nm). The fluorescent intensity was calculated in arbitrary units (AUs). All the captured data were further analyzed with FlowJo version 7.6.5 software (TreeStar, CA, United States).

## Results

### YlxR-Mediated P*frlB* Expression at the *amyE* Locus

**FIGURE 2:**
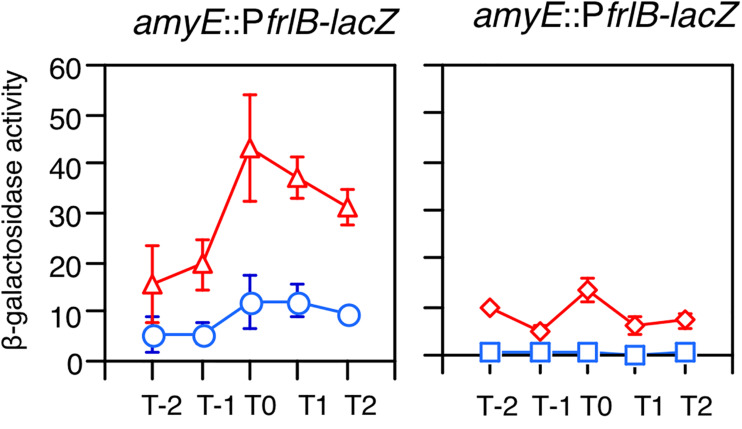
Expression of P*frlB-lacZ.* Strains were grown in sporulation medium and sampled hourly. The *x* axis represents the growth time in hours relative to the end of vegetative growth (T0). Means from three independent experiments and the standard deviations are shown. Left graph: circles, wild type (OAM914); triangles, *ylxR* (OAM915). Right graph: diamonds, *codY* (OAM917); squares, *frlR* (OAM916).

**FIGURE 3:**
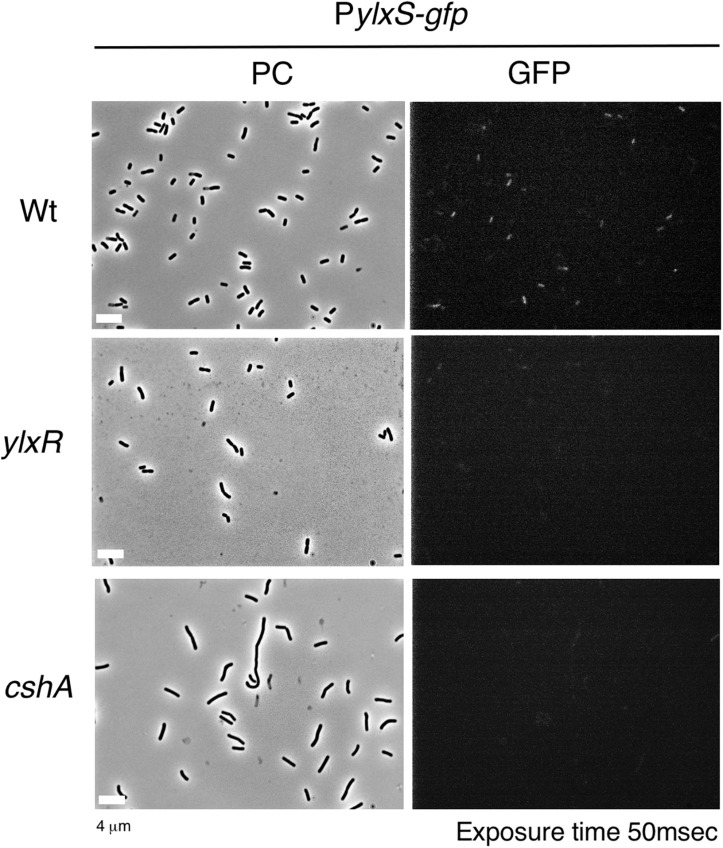
Expression of P*ylxS-gfp.* Strains bearing *amyE*:P*ylxS-gfp*, wild type (OAM818), *cshA* (OAM938), and *ylxR* (OAM939) were grown in LB medium in L-tubes. After 14 h, the cells were sampled and processed. Representative micrographs of the microscopic observation are shown. PC, phase contrast; GFP, green fluorescent protein; LB, Luria–Bertani. GFP fluorescence was visualized using a WIB filter set (Olympus). Image processing and data analysis were performed using Adobe Photoshop CS5.

**FIGURE 4:**
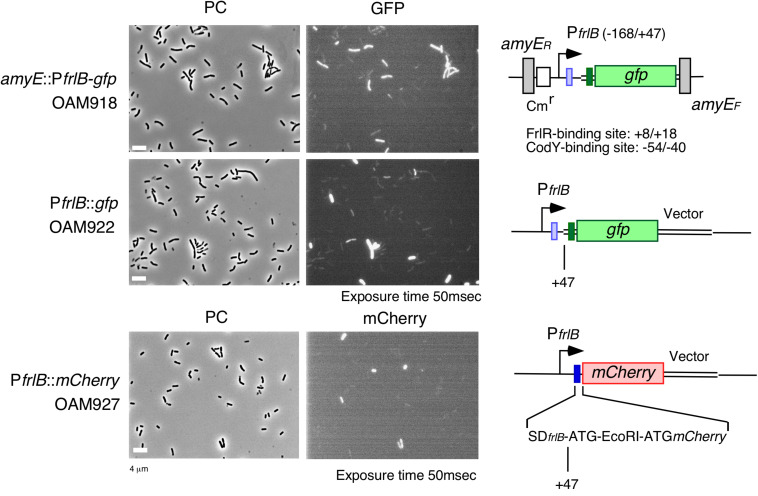
Expression of P*frlB-gfp* fusions located at its own and ectopic chromosomal regions and P*frlB-mCherry.* Structures of two *gfp* and *mCherry* fusions are schematically depicted alongside the corresponding micrographs. The large and small boxes on the line represent the open reading frames and Shine–Dalgarno (SD) sequences (blue for *frlB* and green for *gfp*). The pale box represents an SD sequence that failed to function. The bent arrow indicates the promoter. All the strains retain the intact *frlB* gene. The numbers indicate the nucleotide positions relative to the transcription start nucleotide. Strains were grown in LB medium in L-tubes. PC, phase contrast; GFP, green fluorescent protein; and mCherry, red fluorescent protein derived from *Discosoma* sp. GFP and mCherry fluorescence were visualized using WIB and WIG filter sets (Olympus, Tokyo, Japan), respectively. Image processing and data analysis were performed using Adobe Photoshop CS5. Representative images are shown.

**FIGURE 5:**
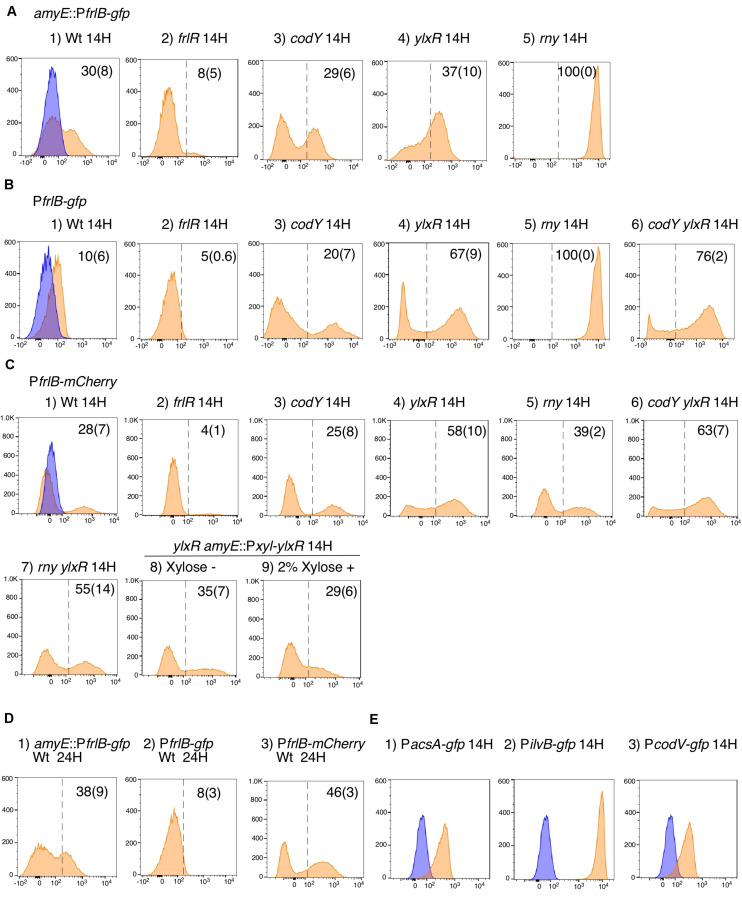
Flow cytometry analysis of three fluorescent P*frlB* fusions. Strains as follows were grown in LB medium. **(A)** *amyE*:P*frlB-gfp*. (1) OAM918; (2) OAM919; (3) OAM920; (4) OAM921; (5) OAM940. **(B)** P*frlB-gfp*. (1) OAM922; (2) OAM923; (3) OAM924; (4) OAM925; (5) OAM941; (6) OAM926. **(C)** P*frlB-mCherry*. (1) OAM927; (2) OAM928; (3) OAM929; (4) OAM930; (5) OAM942; (6) OAM931; (7) OAM943; (8 and 9) OAM944. **(D)** Results of longer cultivation time for three wild-type fusions. **(E)** (1) OAM935; (2) OAM937; (3) OAM936. *X*- and *Y*-axes indicate fluorescence intensity and cell numbers, respectively. The biexponential transformation was applied to display the flow cytometry data and *X*-axis is in “logicle” scale (Parks et al., 2006). In B4 and B6, to show cells with very weak fluorescence intensity, the *X*-axis is expanded to 10^– 3^ but not 10^– 2^. Mean percentages of fluorescence-positive cells from three independent experiments are shown with standard deviations in parentheses. The dotted lines indicate the ends of fluorescence-negative cell fractions (also as blue fractions) obtained from measurement using the control strain 168. Typical patterns are shown. “H” indicates incubation time in hours. LB, Luria–Bertani.

The *frlBONMD-yurJ* operon is subject to the severe YlxR-dependent transcription repression according to previous YlxR-transcriptome analysis (Wiame et al., 2004; Deppe et al., 2011a, b; Ogura and Kanesaki, 2018). To confirm this repression, the P*frlB-lacZ* transcriptional fusion at *amyE* was constructed, and the influence of *ylxR* disruption on P*frlB* expression was measured. Expression of P*frlB-lacZ* was clearly repressed by YlxR as its expression in the *ylxR*-disruptant strain increased four-fold compared with that in the WT strain (Figure 2). We note that sporulation media were used for the *lacZ* and previous transcriptome analysis experiments. With the use of P*ylxS*-*gfp*, no YlxR-expressing (YlxR-ON) cells were observed during growth in SM (Ogura and Kanesaki, 2018); however, YlxR-ON cells are observed 30% of the cell population when grown in LB medium (Figure 3; supporting information in Ogura and Kanesaki, 2018). In addition, heterogeneous *ylxR* expression was subject to positive autoregulation of *ylxR* as disruption of *ylxR* resulted in no expression of P*ylxS-gfp* (Figure 3). This autoregulation is reported to be indirect and mediated by CshA (Figure 1; Ogura and Kanesaki, 2018). This was confirmed by our observation that *cshA* disruption also abolished P*ylxS-gfp* expression (Figure 3). This suggested that YlxR-regulated P*frlB* expression may also be heterogeneous in LB medium. In the previous study of P*frlB*, M9 medium supplemented with Amadori products (fructosamines) was used, and thus growth profile showed diauxie probably due to two carbon sources, glucose and Amadori products (Deppe et al., 2011b). However, we were not able to produce Amadori products efficiently (see section “Discussion”). Thus, to avoid diauxie and difficulties for preparing M9 with Amadori products, we used LB medium for further investigation using fluorescence reporter. To examine possible heterogeneous expression of *frlB*, we constructed a transcriptional P*frlB-gfp* fusion at the *amyE* locus. As shown in Figure 4, the autonomous SD sequence of *frlB* failed to function due to the long distance between the SD sequence and the initiation codon of *gfp*. The strain bearing this fusion showed approximately 30% GFP-ON cells in the microscopic analysis with the remaining 70% being GFP-OFF cells (Figure 4). We note that the expression was observed during early stationary phase of growth in LB medium, i.e., overnight culture. To confirm this observation, flow cytometry analysis of the strain bearing P*frlB-gfp* at *amyE* was performed. As shown in Figures 5A1,_D1, distinct bimodal expression patterns of GFP expression were seen in WT strains after 14 and 24 h of culturing. However, disruption of *ylxR* resulted in a 10% increase in the frequency of GFP-ON cells among the culture population (Figure 5A4). This was consistent with the YlxR repression of the *lacZ* fusion, because total fluorescence increased 2.5-fold. Recently, it has been reported that GFP expression driven by an IPTG-dependent promoter to some extent shows heterogeneous expression, which is due to “noise” in gene expression (Cao and Kuipers, 2018). To examine the expression of other promoters, we randomly selected three (P*acsA*, P*ilvB*, and P*codV*) and created transcriptional GFP fusions, and then we evaluated their expression by flow cytometry. All three of the promoter fusions showed homogeneous expression (Figure 5E). These results indicated that the observed bimodal expression of P*frlB-gfp* was derived from regulated expression specific for P*frlB*, not simply from gene and protein expression “noise.”

According to previous reports, the repressors CodY and FrlR bind to the promoter region of *frlB* (Molle et al., 2003; Belitsky and Sonenshein, 2008; Deppe et al., 2011b). The P*frlB-lacZ* and P*frlB-gfp* at *amyE* constructs that we generated for the current study contained both binding sites. Moreover, disruption of either *codY* or *frlR* has been reported to abate the repression of fusion expression at *amyE* (Belitsky and Sonenshein, 2008; Deppe et al., 2011b). To confirm these observations, we disrupted *frlR* and *codY* in the strains expressing P*frlB-lacZ* and P*frlB-gfp* at *amyE*. Both β-galactosidase (beta-Gal) and flow cytometric analyses showed that the disruption of *frlR* almost completely abolished the expression of both P*frlB-lacZ* and P*frlB-gfp* (Figures 2, 5A2). This suggested that under the conditions we used, FrlR acted on P*frlB* as an activator, not repressor. This was contrary to the previous report by Deppe et al. (2011b). Both the beta-Gal and flow cytometric analyses showed that disruption of *codY* had no detectable influence on the *lacZ* and *gfp* expression (Figures 2, 5A3), again contrary to a previous report, where minimal medium with ammonium or that supplemented with 16 amino acids was used (Belitsky and Sonenshein, 2008). We note that in synthetic medium containing casamino acid (MC medium), basal expression of P*frlB-lacZ* in the WT strain was approximately 10 Miller units, and a slight increase (approximately 2.5-fold) was observed in the *codY* disruptant (data not shown). However, this enhanced rate of the fusion expression was 500-fold lower than the value reported by Belitsky and Sonenshein (2008). We will argue this difference (see section “Discussion”).

### YlxR- and CodY-Mediated P*frlB-gfp* Expression at the Original Chromosomal Locus

In a previous report, expression of P*frlB* (*yurP*)*-lacZ* in its original chromosomal context is 10-fold enhanced by *codY* disruption when cultured in minimal glucose-glutamine medium containing a mixture of 16 additional amino acids (Molle et al., 2003). In the transcriptome (ChIP-to-chip and DNA microarray) analyses using the cells grown in SM, i.e., detection of intact mRNA, 20-fold enhancement of *frlB* mRNA amount was observed by *codY* disruption (Molle et al., 2003). Therefore, we constructed a transcriptional P*frlB-gfp* fusion at its original locus. Heterogeneous expression of the fusion was still observed by microscopic analysis (Figure 4). In the middle row in Figure 4, about 10% of GFP-ON cells were observed. The fusion expression was then analyzed by flow cytometry. The heterogeneous expression of P*frlB-gfp* was observed, but not in a bimodal fashion (Figures 5B1,_D2). The observation of 10% GFP-ON cell fraction was consistent with that in microscopy. As expected, disruption of *frlR* in this strain resulted in almost complete abolishment of P*frlB-gfp* expression (Figure 5B2). In the case of *codY* disruption, the frequency of the GFP-ON cells was increased two-fold (Figure 5B3). It should be noted that average intensity of fluorescence among the GFP-ON cells increased 10-fold in the *codY* disruptant. These findings were consistent with a previous report of approximately a 10-fold enhancement of P*frlB-lacZ* fusion expression (Molle et al., 2003). Disruption of *ylxR* in this strain resulted in a seven-fold larger subpopulation of GFP-ON cells among the population, and the bimodal fashion of expression was restored (Figure 5B4). Our earlier results suggested the possibility that CodY and YlxR may function cooperatively (Ogura and Kanesaki, 2018). To examine this possibility, we constructed a *codY/ylxR* double mutant and measured the frequency of GFP-ON cells among the population. A non-synergistic additive increase was observed, suggesting CodY and YlxR are not in the same regulatory line. Moreover, the frequency of GFP-ON cells was not 100% in the double mutant, suggesting that an unknown factor may function in this double mutant (Figure 5B6, see below, section on RNaseY).

### YlxR-Mediated P*frlB-mCherry* Translational Fusion Expression at the Original Locus

As detailed in the Supplementary Table S1 in Ogura and Kanesaki (2018), YlxR regulates the expression of more than 400 genes, including translation-related genes such as *tsaD* (encodes a component of the enzyme required for t^6^A modification of tRNA), *rrnE*-16S (encodes an rRNA), and *rpsNB* (encodes ribosomal protein S14). Therefore, it is possible that YlxR acts not only on the transcription of P*frlB* but also on its translation. To explore this possibility, we constructed a translational fusion of *frlB* with the fluorescence protein gene *mCherry* at its original chromosomal position. In the construct, the mCherry protein was added with three amino acids to the N-terminus and expressed with the *frlB* SD sequence as required for its translational initiation (Figure 4). Microscopic analysis revealed heterogeneous expression of P*frlB-mCherry*, as expected (Figure 4). In flow cytometric analysis, a subpopulation of P*frlB*-mediated mCherry-ON cells was represented by a distinct peak, indicating bimodal expression of mCherry (Figures 5C1,_D3). We note that a higher rate of mCherry-ON cells was detected by flow cytometry than that by microscopic analysis. Rapid fading of mCherry with an N-terminal adduct (a half-life of approximately 3 seconds according to the analysis of continuous photographing of fluorescence images; data not shown) resulted in more efficient detection of fluorescence by flow cytometry. The distinctly different expression profiles between transcriptional P*frlB-gfp* and translational P*frlB-mCherry* fusions, both of which are located at its original locus, suggested regulation other than transcriptional regulation, that is, at the post-transcriptional level including translation. Disruption of *ylxR* in the strain bearing the *mCherry* fusion caused severe decline of the sharp bimodal expression, suggesting that *frlB*-mediated bimodal expression of *mCherry* required functioning YlxR (Figure 5C4). To confirm this, a complementation test of *ylxR* disruption by xylose-inducible *ylxR* was performed. Without xylose, frequency and expression profile of mCherry-ON cells were similar to those in the WT, suggesting complementation of *ylxR* disruption probably due to leaky expression of P*xyl*-*ylxR* (Figure 5C8). Further induction of *ylxR* with xylose showed slightly decreased frequency of mCherry-ON cells and reduced total fluorescence of the mCherry fusion to 30% of that in the absence of xylose, leading to change of the expression profile (Figure 5C9). A cell population with fluorescence intensity more than 10^3^ almost disappeared by addition of xylose (Figure 5C8 vs. 5C9). These experiments showed that the severe decline of the sharp bimodal expression is indeed caused by the *ylxR* disruption, but not polar effect. It should be noted that in the *ylxR* disruptant, still significant mCherry-OFF cells were observed, which suggested the uncovered regulatory mechanism in the *frlB* expression. Disruption of *frlR* also almost completely abolished the *mCherry* expression, as expected (Figure 5C2), but disruption of *codY* had no noticeable effect on *mCherry* expression, suggesting a negligible if any increase in transcription of *mCherry* in the *codY* disruptant (Figure 5B vs. 5C). Disruption of *codY* in association with the disruption of *ylxR* enhanced frequency of mCherry-ON cells slightly, compared with that seen for the disruption of *ylxR* alone (Figure 5C6). This suggested that the enhancement of transcription due to the disruption of *codY*, as observed in the case of P*frlB-gfp* located at its original locus, did not result in the higher frequency of mCherry-ON cells among the population than that in the single *ylxR* disruptant.

### *frlB* Expression in the *rny* Depletion Mutant

Expression of P*frlB* is reported to be negatively regulated by RNaseY, which is encoded by *rny* and has endonuclease activity for many mRNAs (Lehnik-Habrink et al., 2011). That cleavage triggers mRNA degradation. Although the entire mRNA structure between *frlB* itself and P*frlB-gfp* transcriptional fusions is different, those mRNAs share the common short upstream untranslated region in structure (Figure 4). Therefore, expression of P*frlB-gfp* fusion at either *amyE* or the original locus may be increased in the *rny*-depleted strain. Consistent with this expectation, we observed 100% GFP-ON cells from the population among the two strains bearing both P*frlB-gfp* and *rny*-depletion (Figures 5A5,_B5). These findings indicated that RNaseY acted through the degradation of P*frlB*-driven mRNA and was perhaps responsible for the bimodal expression profiles of these fusions. Consequently, the depletion of *rny* may not have affected *frlB* expression at the level of translation. To examine this, the *rny* depletion was introduced into a strain bearing P*frlB-mCherry*, and the expression was evaluated in flow cytometric analyses. As expected, the depletion of *rny* only slightly affected the bimodal expression of P*frlB-mCherry*, i.e., 10% enhancement of frequency of mCherry-ON cells (Figure 5C5). This indicated that the depletion of *rny* affected the expression of *frlB* transcriptional fusions profoundly, but not significantly its translational control. Moreover, the frequency of mCherry-ON cells in the *rny* depleted strain with the *ylxR* disruption was similar to that in the *ylxR* single disruptant (Figure 5C7). This result is also consistent with the observation that RNaseY did not affect translational *frlB* fusion expression.

### Microscopic Analysis of Cells Bearing P*frlB-mCherry* Translational Fusion and *amyE*:P*ylxS-gfp*

**FIGURE 6:**
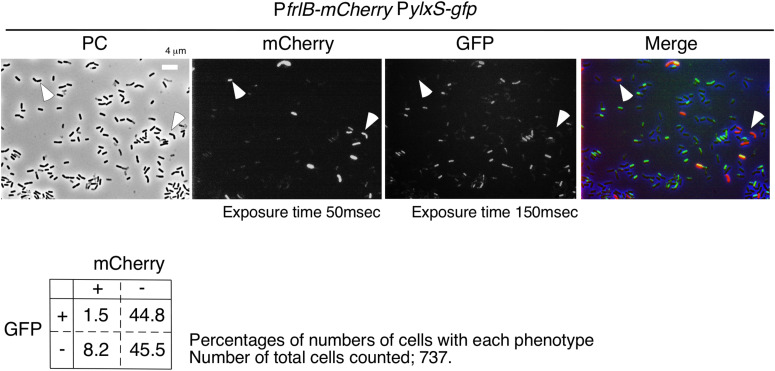
Expression of *gfp* and *mCherry* in cells bearing P*ylxS-gfp* and P*frlB-mCherry.* OAM932 was grown in LB medium in L-tubes. After 14 h of incubation, cells were sampled and processed. Representative micrographs from the microscopic observation are shown. PC, phase contrast; GFP, green fluorescent protein; mCherry, red fluorescent protein; LB, Luria–Bertani. GFP and mCherry fluorescence were visualized using WIB and WIG filter sets (Olympus), respectively. Arrowheads indicate mCherry-ON/GFP-OFF cells. Image processing and data analysis were performed using Adobe Photoshop CS5. The merged micrographs are in shown with pseudocolor, red, mCherry; green, GFP. Results of the quantitative analysis are shown below the photos. Chi-square test of independence was performed to compare the frequencies or proportions among variables in four types of cells with respect to mCherry and GFP. Chi-square value, *p*-value, and degrees of freedom (df) value were 30.19, 3.93 × 10^– 8^, and 1, respectively. This indicated that the chi-square statistic was at a significant level.

Flow cytometric analysis suggested that YlxR mediated negative control of P*frlB-mCherry*. Moreover, as YlxR expression is heterogeneous (Ogura and Kanesaki, 2018), P*frlB-mCherry*-expressing cells may be differentiated from YlxR-OFF cells. To examine this possibility, we constructed the strain with P*ylxS-gfp* at *amyE* and P*frlB-mCherry*. Unfortunately, P*ylxS-gfp* fluorescence was very weak, resulting in flow cytometric analysis detecting only a few percent of GFP-ON cells among the population of cells (0.5–5% in three independent observations), which was lower than that observed in the microscopic analysis. This may be due to detection of auto-fluorescence derived from the intracellular molecules like NADPH and aromatic amino acids from cells with no GFP-expressing strain, i.e., the control strain 168, in flow cytometry. Discrimination of weak but significant fluorescence signals from auto-fluorescence is difficult in flow cytometry. Contrary to this, in microscopic analysis, we did not observe auto-fluorescence derived from the strain without the *gfp* gene under the conditions we used (data not shown). Therefore, we had to explore the possibility using the microscopic analysis. As shown in Figure 6, most mCherry-ON cells lacked P*ylxS-gfp* expression; however, a small fraction of mCherry-ON cells did exhibit associated P*ylxS-gfp* expression. Among the mCherry-ON cells, distribution of P*ylxS-gfp* expression was low compared with that among the mCherry-OFF cells with the difference being statistically meaningful (see legend to Figure 6). These results suggested that the mCherry-ON cells were often differentiated from cells that did not expressed YlxR. This was consistent with the results obtained from the flow cytometric analysis.

## Discussion

**FIGURE 7:**
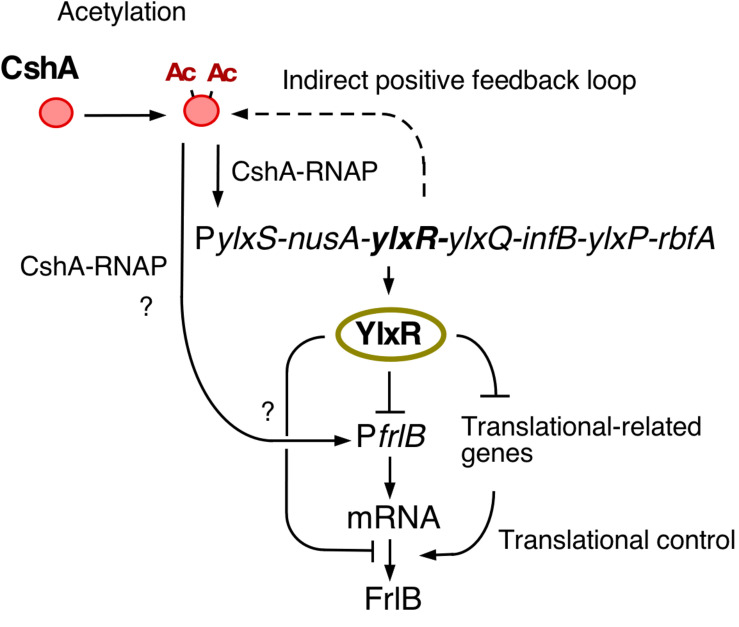
Hypothetical schematic for the regulation of *frlB* expression. Arrows and T-bars indicate positive and negative actions, respectively. Ac, acetyl moiety; RNAP, RNA polymerase.

In this study, we observed bimodal expression of P*frlB-mCherry*. Gene products of the *frlB* operon are used for the utilization of amino sugars. However, to the best of our knowledge, there are no reports on whether sporulation or LB media contain amino sugars. In synthetic MC medium, which does not contain amino sugars, *frlB* expression did not change compared with that in sporulation or LB media (Ogura, unpublished results). These results indicated that some cells differentiate into P*frlB*-expressing cells, even though there is no availability of amino sugars. This means that the *Bacillus* cells adopt a bet-hedging strategy with respect to nutritional fluctuation. At the transcription level, the observed bimodal or heterogeneous expression of P*frlB* located at ectopic or original locus was caused by mRNA degradation triggered by RNaseY; however, this regulation was restricted for the transcriptional fusion expression through mRNA stability control. Based on our current study, we suggest that YlxR bimodal expression may result in the bimodal expression of the *frlB* operon through regulation at both the transcription and post-transcription levels including translation. Similar to YlxR in *Bacillus subtilis*, *Escherichia coli* nucleoid-associated proteins Ler and H-NS cause bimodal expression from the enterocyte effacement (LEE) pathogenicity loci (Leh et al., 2017). The exact mechanism underlying *ylxR* bimodal expression is not known. Generally, positive feedback regulation is responsible for the bimodal expression of a gene (Veening et al., 2008; Norman et al., 2015; Bury-Moné and Sclavi, 2017). Our previous reports showed that YlxR is subject to an indirect positive feedback loop (Ogura and Asai, 2016; Ogura and Kanesaki, 2018; Ogura et al., 2019). This loop may be responsible for the bimodal expression of YlxR (Figures 1, 7).

Disruption of *codY* enhanced the expression of the transcriptional P*frlB-gfp* fusion but did not affect the expression of the translational fusion. Considering the changes in the expression profiles between both fusions also suggested the regulation of *frlB* was at the post-transcriptional level including translation. Two possible routes of YlxR-dependent translational regulation of *frlB* were feasible (Figure 7). First, YlxR itself may function in *frlB* translation. It is possible that YlxR binds to *frlB* mRNA and thereby affects its translation as the crystal stereo-structure of YlxR suggests RNA-binding by YlxR (Osipiuk et al., 2001). Second, the YlxR-regulon contains several translation regulatory factors, including *rrnE*-16S and *rpsNB*, whose expression is directly or indirectly repressed by YlxR (Ogura and Kanesaki, 2018). Thus, disruption of *ylxR* enhances the expression of these genes. This may lead to the activation of *frlB* translation, which would then result in the disruption of the distinct bimodal expression profile of *frlB*.

The expression of *ylxR* is dependent on *cshA*, and therefore, disruption *cshA* would be expected to increase *frlB* expression as the *cshA* disruption would lead to decreased expression of YlxR, the negative regulator of *frlB* (Figures 1, 7). However, according to a previous transcriptome analysis of the *cshA* disruptant, the *frlB* operon is severely suppressed (Lehnik-Habrink et al., 2013). We also observed a significant decrease in the expression of *frlB* in the preliminary RNA-seq analysis using the *cshA* disruptant with approximately a 70% reduction compared with that in the WT strain (Ogura and Kanesaki, unpublished results). In addition, flow cytometry analysis using P*frlB-gfp* at *amyE* revealed no GFP fluorescence expression in the *cshA* mutant (Ogura, unpublished results). Consequently, we speculated that *cshA* may positively regulate the expression of *frlB*, independent of *ylxR* (Figure 7). This should be clarified in future analyses.

A previous study presented a model where FrlR was a repressor and suggested that the inducer fructosamine-6-phosphate may inhibit the repressor activity of FrlR, leading to expression of the *frlB* operon (Deppe et al., 2011b). Our experimental data are inconsistent with that model. First, FrlR acted on the expression of P*frlB* as an activator, not a repressor. FrlR belongs to the GntR bacterial transcription family, and that family includes several transcriptional activators (Blancato et al., 2008; Wiethaus et al., 2008; Edayathumangalam et al., 2013; Brambilla and Sclavi, 2015), which reinforces our conclusion. Second, in the previous study one of Amadori products, fructose-arginine was synthesized and used to show that it was an inducer of the *frlB* operon through acting on FrlR (Deppe et al., 2011b). The protocol for the synthesis of fructose-arginine in the previous paper is ambiguous, and we therefore used a modified procedure. Nuclear magnetic resonance spectroscopy analysis of the reaction products indicated small but significant amounts of reaction products (Shindo, unpublished results). However, we added the reaction products to culture media of strains with both types of the P*frlB-gfp* fusions, which resulted in no influence on *gfp* expression (Ogura, unpublished results). Based on these data, we concluded that FrlR functions as an activator and that fructose-arginine is not an inducer of P*frlB* expression at least in LB medium.

We observed that CodY only functioned when the target *frlB* promoter was located in its original chromosomal position as disruption of *codY* did not affect *frlB* expression from the *amyE* locus. However, in an earlier report, P*frlB-lacZ* expression at *amyE* increased in the *codY* disruptant (Belitsky and Sonenshein, 2008). The fusion used in the earlier report bears the longer promoter region of *frlB* (-202/ + 90) than that used in this study (-168/ + 47). The difference may have led to the discrepancy in the results. Under the conditions we used, however, *codY* disruption influenced *frlB* expression at its original position, but not its ectopic *amyE* position. Thus, the function of CodY might be dependent on the chromosomal position of the target gene *frlB*. This is not unprecedented as several other examples have been reported (Bryant et al., 2014; Brambilla and Sclavi, 2015).

There are few known cases of bimodal expression of metabolic genes. In *E. coli*, when cells are transferred from glucose-containing media to media with a different carbon-source medium, such as malate, which is used for gluconeogenesis, most of the cells remain dormant in a persister state; however, a subpopulation that is prepared to use malate and the gluconeogenesis pathway appears among the cell population (Kotte et al., 2014). In *Pseudomonas putida*, when glycerol is the sole carbon source, bistable expression of the glycerol utilization operon occurs due to the repressor of that operon (Nikel et al., 2015). These are examples of metabolic bet-hedging strategies. In addition, when cells were grown using some carbon-source, such as D-xylose for *E. coli* and myo-inositol for *Salmonella*, expression of the operon promoters for their utilization is bimodal (Kröger et al., 2011; Afroz et al., 2014). In *Lactococcus lactis*, carbon diauxie (glucose to cellobiose) results in the bimodal expression from the promoter of the genes encoding the sugar phospho-transfer system (PTS) for cellobiose/lactose (Solopova et al., 2014). Interestingly this bimodal expression during diauxie disappears with the disruption of CcpA, which is a master regulator of carbon catabolites in gram-positive bacteria and represses that PTS promoter (Fujita, 2009). Therefore, bimodal expression of *frlB* in *B. subtilis* deserves further study, since the substrate of FrlB is not the most favorable carbon source but is sometimes provided. For example, this is the case in rhizospheres where it is actually preferred by Gram-positive bacteria of the family *Bacillaceae*.

## Data Availability Statement

The original contributions presented in the study are included in the article/Supplementary Material. further inquiries can be directed to the corresponding author.

## Author Contributions

MO performed experiments and wrote the manuscript. KS performed experiments. YK performed statistical analyses. All authors contributed to the article and approved the submitted version.

## Conflict of Interest

The authors declare that the research was conducted in the absence of any commercial or financial relationships that could be construed as a potential conflict of interest.
