# Supplementary material for: Bacillus subtilis Nucleoid-Associated Protein YlxR Is Involved in Bimodal Expression of the Fructoselysine Utilization Operon (frlBONMD-yurJ) Promoter
Source: Front Microbiol. 2020 Aug 21;11:2024. doi: 10.3389/fmicb.2020.02024 (PMC7475707; doi:10.3389/fmicb.2020.02024)
Supplement: Supplementary file 1 [file Data_Sheet_1.PDF]

Table S1. Oligonucleotides used for this study.

| Name | Sequence |
|---|---|
| pIS-frlB-F-E | 5'-ACGGAATTCGACGTCAGTCTGCGGATTG-3' |
| pIS-frlB-R-B | 5'-ACGGGATCCATCCTTCACTCCTCGTTTTTA-3' |
| pIS-acsA-F-E | 5'-ATTGAATTCTTCCGACAGCGCATCCCCGA-3' |
| pIS-acsA-R-B | 5'-TTCGGATCCTCTGCCTCGGCCCAATCAAAA-3' |
| pIS-codV-Eco | 5'-ATCGAATTCAGGAAACAGCAATTGGCAG-3' |
| pIS-codV-Bam | 5'-ATCGGATCCTAATACCTCCCCAGGAAGG-3' |
| PfrlB-(SD)-gfp-R | 5'-GTAGTTCCTCCTTATGATCCTTCACTCCTCGTTTTTA-3' |
| PacsA-(SD)-gfp-R | 5'-GTAGTTCCTCCTTATGTCTGCCTCGGCCCAATCAAAA-3' |
| PcodV-(SD)-gfp-R | 5'-GTAGTTCCTCCTTATGCCTAATACCTCCCCAGGAAGG-3' |
| PilvB-(SD)-gfp-R | 5'-GTAGTTCCTCCTTATGGTGAAGCTTGCATTTATCTT-3' |
| amyE-RR | 5'-TCAATGGGGAAGAGAACCGCTTAAGCCCG-3' |
| amyE-FF | 5'-GTTAACAAAATTCTCCAGTCTTCACATCGG-3' |
| gfp(SD)-F | 5'-CATAAGGAGGAACTACTATGAGTAAAG-3' |
| gfp-Xba-R | 5'-GCTCTAGATTATTTGTATAGTTCATCCATGCCA-3' |
| mChe-frlB-F-H | 5'-TTGAAGCTTCACCGATCAAACATCACAG-3' |
| mChe-frlB-R-E | 5'-ACGGAATTCCATATCCTTCACTCCTCGTTTTTA-3' |
| pUKM-frlR-B | 5'-TTCGGATCCGGTTAAAAGGAGGCTACCCT-3' |
| pUKM-frlR-H | 5'-TTGAAGCTTTTCGGCGATGGG-3' |
